# A new therapy against ulcerative colitis via the intestine and brain using the Si-based agent

**DOI:** 10.1038/s41598-022-13655-7

**Published:** 2022-06-10

**Authors:** Yoshihisa Koyama, Yuki Kobayashi, Ikuei Hirota, Yuanjie Sun, Iwao Ohtsu, Hiroe Imai, Yoshichika Yoshioka, Hiroto Yanagawa, Takuya Sumi, Hikaru Kobayashi, Shoichi Shimada

**Affiliations:** 1 Department of Neuroscience and Cell Biology, Osaka University Graduate School of Medicine, 2-2 Yamadaoka, Suita, Osaka 565-0871 Japan; 2 Addiction Research Unit, Osaka Psychiatric Research Center, Osaka Psychiatric Medical Center, Osaka, 541-8567 Japan; 3 SANKEN, Osaka University, Osaka, 567-0047 Japan; 4 University of Tsukuba, Faculty of Life and Environmental Sciences, 108-2, Cooperative Research Building A, Ibaraki, 305-8577 Japan; 5 Euglena Co., Ltd., Tokyo, 408-0014 Japan; 6 University of Tsukuba, R&D Center for Tailor-Made-QOL, 108-2, Cooperative Research Building A, 1-1-1 Tennodai, Tsukuba, Ibaraki 305-8577 Japan; 7 Graduate School of Frontier Biosciences, Osaka University, Osaka, 565-0871 Japan; 8 Center for Information and Neural Networks, National Institute of Information and Communications Technology (NICT) and Osaka University, Osaka, 565-0871 Japan; 9 Institute for Open and Transdisciplinary Research Initiatives, Osaka University, Osaka, 565-0871 Japan; 10 Department of Cell Biology, Graduate School of Medicine, Osaka University, Osaka, 565-0871 Japan

**Keywords:** Ulcerative colitis, Metabolomics

## Abstract

Ulcerative colitis (UC) is a non-specific inflammatory bowel disease that causes ulcers and erosions in the colonic mucosa and becomes chronic with cycles of amelioration and exacerbation. Because its exact etiology remains largely unclear, and the primary therapy is limited to symptomatic treatment, the development of new therapeutic agent for UC is highly desired. Because one of the disease pathogenesis is involvement of oxidative stress, it is likely that an appropriate antioxidant will be an effective therapeutic agent for UC. Our silicon (Si)-based agent, when ingested, allowed for stable and persistent generation of massive amounts of hydrogen in the gastrointestinal tract. We demonstrated the Si-based agent alleviated the mental symptom as well as the gastrointestinal symptoms, inflammation, and oxidation associated with dextran sodium sulfate-induced UC model through Hydrogen and antioxidant sulfur compounds. As the Si-based agent was effective in treating UC in the brain and large intestine of mice, it was considered to be capable of suppressing exacerbations and sustaining remission of UC.

## Introduction

Ulcerative colitis (UC), an inflammatory bowel disease (IBD), is a cryptogenic chronic disease that causes erosions or ulcers due to inflammation of the mucous membrane of the large intestine^1^. Although its exact etiology has not yet been fully elucidated, it is accepted that the onset of UC occurs due to an inappropriate aggressive inflammatory response. Since the primary treatment is limited to symptomatic treatment, the development of new therapeutic agent is highly desired.

One of UC pathogenesis is the oxide accumulation in the intestinal mucosa caused by reactive oxygen species (ROS), which have a strong oxidizing effect^2^. The accumulation of such oxides affects various transcriptional activities and phosphorylation signals, thereby causing inflammation. In particular, an increase in lipoperoxide (LPO) levels damages the endothelial cells in the large intestine and exacerbates the symptoms of colitis, which can develop into colon cancer^3^. In fact, endogenous antioxidant enzymes influence the severity of colitis symptoms. Glutathione peroxidase (GPx)-deficient mice spontaneously develop colitis^4^. Further, in some studies, in mice administered super oxide dismutase (SOD)^5^ or catalase^6^ in vivo and in SOD-overexpressing mice^7^ colitis-associated symptoms were found to be alleviated. Thus, oxidative stress is greatly involved in the onset and exacerbation of colitis, and it is likely that an appropriate antioxidant could serve as an effective therapeutic agent for UC.

In 2007, molecular hydrogen was reported to be an antioxidant that can specifically neutralize harmful ROS, hydroxyl radicals^8^. It is expected to be an especially useful antioxidant, as it shows excellent permeability and has no side effects. Since its discovery, the effectiveness of molecular hydrogen in various diseases, such as ischemia–reperfusion injury^8^, IBD^9^, and hepatitis^10^ has been reported.

Our silicon (Si)-based agent generated hydrogen in response to water. As the direct reaction species to react with Si-based agent is hydroxide ions, the higher the pH of the reaction solution, the greater the amount of hydrogen generated from Si-based agent. The duration of hydrogen generation has also been reported to be long^11^. Therefore, it is possible to continuously generate a large amount of hydrogen in the large intestine, in which the environment is neutral to weakly alkaline, by oral administration of the Si-based agent. In fact, treatment with an Si-based agent suppresses both the development of renal failure and the decrease in motor coordination in Parkinson's disease^12^. Keeping the large intestine filled with antioxidants may be an effective means to alleviate the symptoms of UC, because the large intestine is the primary inflammation site.

The dextran sodium sulfate (DSS)-induced colitis animal model in IBD research has several advantages over other chemically induced experimental models owing to its rapidity, simplicity, and reproducibility^13^. Hence, we investigated the therapeutic effect of our Si-based agent against UC using a DSS-induced UC model.

## Results

### Si-based agent showed efficacy in relieving the symptoms of UC

**Figure 1:**
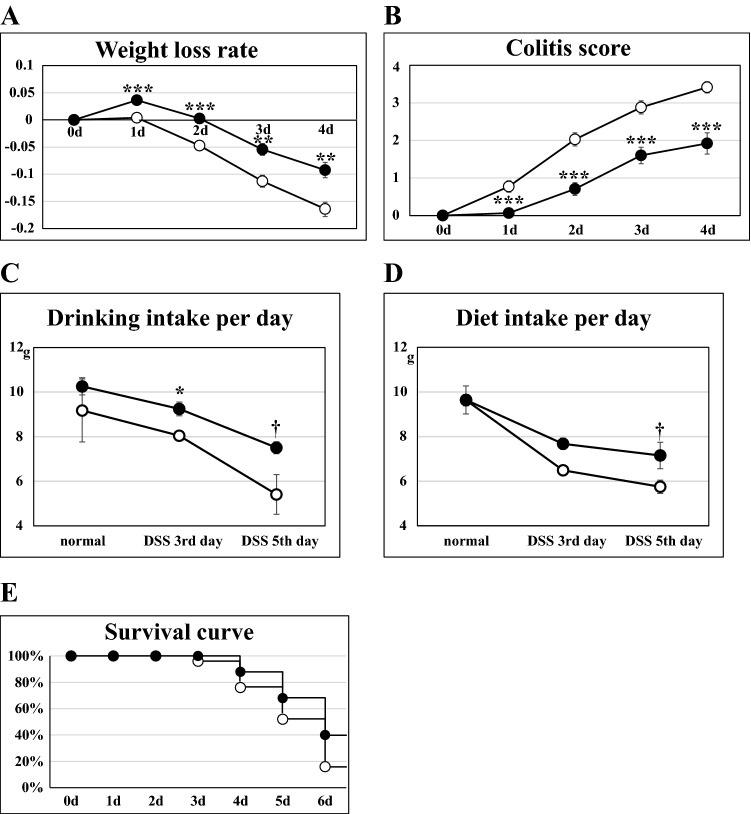
Si-based agent alleviated the symptoms of UC. Time-dependent changes in the gross pathology, the intake of drinking and diet consumed per day, and survival rates of 5% DSS-treated mice. Line chart of weight loss rate (**A**), colitis score (**B**), the intake of drinking (**C**) and diet (**D**) consumed per day, and survival curve (**E**). White: Con-DSS group. Black: Si-DSS group. Each day of the horizontal axis is post-DSS dosing (**A**,**B**,**E**). Each diet treatment for a week (normal), DSS treatment for 3 days (DSS 3rd day) or 5 days (DSS 5th day) after each diet treatment for a week (**C**,**D**). Data are expressed as mean ± SEM of 25 mice (**A**,**B**,**D**), 4 cages (**C**,**D**: normal; 3 mice per cage) and 3 cages (**C**,**D**: DSS 3rd and 5th days) per group. ^†^*p* < 0.09, **p < 0.01, ***p < 0.001 vs. Con-DSS, determined by Wilcoxon rank sum test (**A**,**B**) and Student’s paired *t*-test (**C**,**D**).

To examine whether the Si-based agent prevents the worsening of UC, we generated 5% DSS-induced UC model mice and performed a comparative analysis between the UC model mice fed with 2.5% Si-based agent-containing diets (Si-DSS group) and those fed with diets without the Si-based agent (Con-DSS group). In the Con-DSS group, the weight loss associated with UC was evident on the 2nd day of DSS administration; it further reduced to − 16.38 ± 0.008% on the 4th day (Fig. 1A). In contrast, in the Si-DSS group, the weight increased on the 1st day after DSS administration and then decreased after the 3rd day; the weight loss was -9.24 ± 0.007% on the 4th day (Fig. 1A). The weight loss in the Si-DSS group was significantly suppressed compared with that in the Con-DSS group.

Subsequently, the degree of inflammatory symptoms was examined based on the colitis score. Inflammation in the Con-DSS group progressed from the 1st day of DSS administration; the colitis score was 3.41 ± 0.15 on the 4th day. Contrastingly, inflammation in the Si-DSS group was evident from the 2nd day, and the score was 1.92 ± 0.28, even on the 4th day. The inflammation symptoms in the Si-DSS group were significantly relieved compared with those in the Con-DSS group (Fig. 1B). Since the progression of colitis may also affect the intake of diet and drinking, the intake of diet and drinking consumed per day before and after administration of DSS was measured. In both groups, the intake of diet and drinking consumed per day gradually decreased after DSS administration increased (Fig. 1C,D). However, the decrease in intake tended to be mitigate in the Si-DSS group as compared with the Con-DSS group. No difference was observed between the two groups in the normal intake of diet and drinking consumed per day (Fig. 1C,D).

In addition, mortality associated with UC exacerbation was compared between the two groups. In the Con-DSS group, mice were observed for death from the 3rd day of DSS administration, and the survival rate was found to be 16% on the 6th day. In contrast, animals in the Si-DSS group were observed for death after the 4th day, and the survival rate was 40%, even on the 6th day (Fig. 1E). Taken together, these findings suggest that alleviation of the weight loss and inflammatory exacerbation associated with UC resulted in a significant decline in mortality in the Si-DSS group compared with that in the Con-DSS group (Table 1).

**Table 1:** Coliti score. Add the above 3 values and divide by 3. 0: Healthy, 4: Colitis maximum active state.

	Weight loss	Feces consistency		Anal bleeding
**Colitis score**				
0	0%	Normal formed feces	No feces attached to anus	None
1	1–5%			
2	5–10%	Unformed mucoid feces	No feces attached to anus	Microscopic bleeding
3	10–20%			
4	More than 20%	Liquid stool	Feces attached to anus	Anal hemorrhage

### Administration of the Si-based agent significantly alleviated UC inflammation symptoms

**Figure 2:**
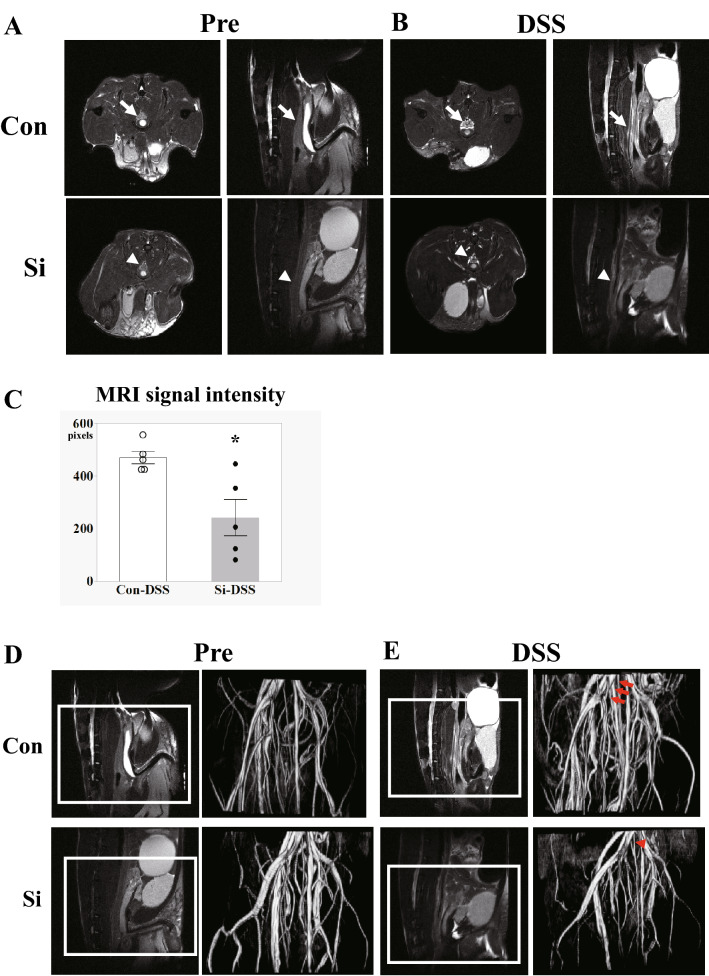
Si-based agent mitigated the inflammation of large intestine. Analysis of inflammation in the colon, using MRI and MRA. (**A**,**B**) Representative T_2_ weighted axial (left) and sagittal images (right) of the colon in the control (upper) and Si-based agent-treated groups (bottom) before treatment (**A**) and 5 days after 5% DSS treatment (**B**). *Pre* pretreatment, *DSS* 5 days after DSS treatment, *Con* control group, *Si* Si group. The colon of a control mouse (arrow) and an Si-based agent-treated mouse (arrowhead). (**C**) Average number of high signal intensity pixels in the axial image of the colon at the level of the pubis. White: Con-DSS group. Black: Si-DSS group. Data are expressed as mean ± SEM of five mice per group. *p < 0.05 vs. Con-DSS, determined by Student’s paired *t*-test. (**D**,**E**) Representative T_2_ weighted sagittal images of the colon (left) and images of blood vessels around the colon (right) in the control (upper) and Si-based agent-treated groups (bottom) before treatment (**D**) and 5 days after DSS treatment (**E**). (**D**,**E**) The colon of a control mouse (arrow) and an Si-based agent-treated mouse (arrowhead). Square: the subject of the blood vessel image around the colon. The abdominal aorta of a control mouse (arrow) and an Si-based agent-treated mouse (arrowhead).

To examine whether inflammation of the large intestine, the primary symptom of UC, was suppressed by administration of the Si-based agent, MRI of the UC model mice was performed on the 5th day after DSS administration. No pixels with high signal intensity were observed in either group under normal conditions (Fig. 2A). While some high signal intensity pixels indicating inflamed edematous regions surrounding the large intestine, were detected in the Con-DSS group, these pixels were scattered in the large intestine and slightly detected in the Si-DSS group (Fig. 2B). The inflammatory signal region in the Si-DSS group was significantly lower than that in the Con-DSS group (Fig. 2C). In addition, the blood vessels in the abdominal aorta, supplying blood to the large intestine and its branch arteries, were analyzed using MRA. Although swelling of the abdominal aorta, which is a characteristic feature of inflammation, was observed in the Con-DSS group on the 5th day after DSS administration, there were no significant changes in the aorta before and after DSS administration in the Si-DSS group (Fig. 2D,E). Imaging analyses demonstrated that the Si-based agent suppressed the inflammation of the large intestine.

**Figure 3:**
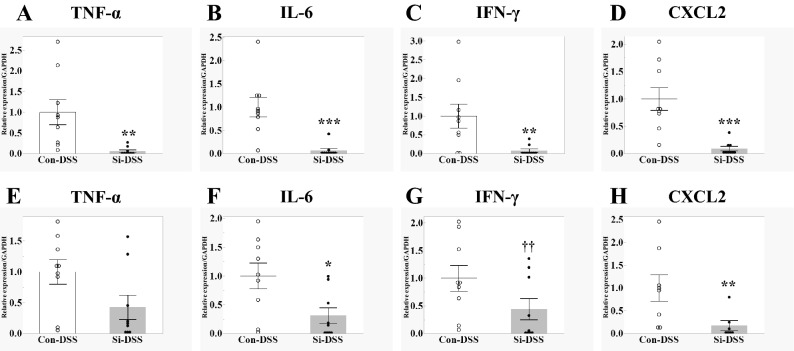
Si-based agent suppressed the increase in pro-inflammatory cytokine and chemokine. mRNA expression of proinflammatory cytokines in the mouse large intestine 3 days after 5% DSS treatment, measured using quantitative reverse transcription-PCR analysis. Bar chart indicates the mean values (rectum: **A**–**D**; colon: **E**–**H**). TNF-α (**A**,**E**), IL-6 (**B**,**F**), IFN-γ (**C**,**G**) and CXCL2 (**D**,**H**). White: Con-DSS group. Black: Si-DSS group. Data are expressed as mean ± SEM of nine mice per group. ^††^*p* < 0.08, *p < 0.05, **p < 0.01, ***p < 0.001 vs. Con-DSS group, determined by Wilcoxon rank sum test.

Next, we examined whether the Si-based agent also affected the immune system involved in colitis. To analyze the RNA expression levels of pro-inflammatory cytokines and chemokines in the rectum, which is the primary UC lesion site, qRT-PCR was performed on the 3rd day after DSS administration. In the Con-DSS group, the expression levels of pro-inflammatory cytokines (TNF-α, IL-6, and IFN-γ) and those of neutrophil-attracting chemokine CXCL2 were increased by colitis. However, the above-mentioned increase in pro-inflammatory cytokine and chemokine RNA expression was significantly suppressed in the Si-DSS group (Fig. 3A–D). Since colon inflammation indicated aggravation of UC, pro-inflammatory cytokines in the colon were also investigated. Although there was no statistically significant difference between the control DSS and Si-DSS groups, it was found that the expression of inflammatory cytokines in the colon was also suppressed by the Si-based agent. In addition, the expression of CXCL2 was significantly suppressed in the colon (Fig. 3E–H).

These results suggest that administration of the Si-based agent significantly alleviated UC inflammation symptoms via the inhibition of pro-inflammatory humoral factor expression.

### Administration of the Si-based agent significantly suppressed colonic atrophy and structural collapse associated with UC

**Figure 4:**
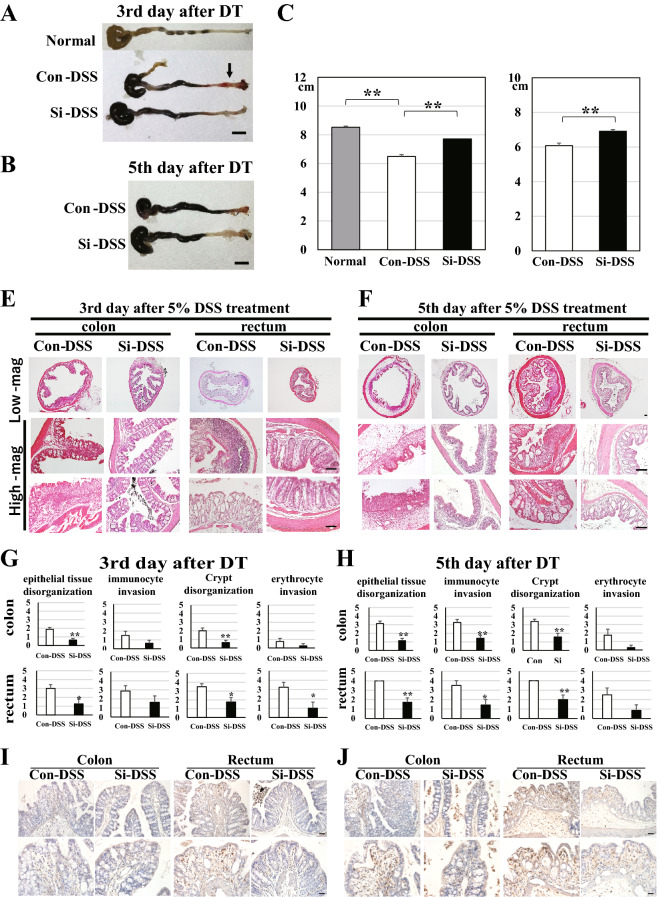
Si-based agent suppressed the atrophy and structural disorder associated with intestinal inflammation. Measurement and pathological analysis of the large intestine of 5% DSS-treated mice. (**A**–**D**) The representative photograph (**A**,**B**) and the bar chart indicate the average of the actual values (**C**,**D**) 3 days (**A**,**C**) and 5 days after DSS treatment (**B**,**D**). (**E**,**F**) The representative HE-staining photographs obtained 3 days (**E**) and 5 days after 5% DSS treatment (**F**). Low-mag: low magnification and High-mag: high magnification. (**G**,**H**) The average bar chart of each histological score 3 days (**G**) and 5 days after DSS treatment (**H**). DT: DSS treatment. Colitis: DSS treatment. Gray: normal mice (4 mice), white: Con-DSS group (8 mice), and black: Si-DSS group (3 days: 8 mice, 5 days: 7 mice). Scale bar: 1 cm (**A**,**B**), 100 µm (**C**,**D**). (**I**,**J**) The representative F4/80-immunostaining photographs obtained 3 days (**I**) and 5 days after 5% DSS treatment (**J**). Upper panels: low magnification and bottom panels: high magnification. Scale bar: 100 µm (**I**), 50 µm (**J**). Data are expressed as mean ± SEM. *p < 0.05, **p < 0.01 vs*.* normal mice or Con-DSS, determined by Student’s paired *t*-test.

Histological analysis was performed to examine whether administration of the Si-based agent is effective in suppressing the injury of the large intestine associated with colitis. In the UC model mice, it has been reported that the length of the large intestine is reduced due to inflammation; hence, we measured the length of the large intestine from the ascending colon to the anus at the rectum. Comparison of the results with the actual values of the large intestine of normal mice revealed that the large intestine of the Con-DSS group was significantly atrophied due to inflammation on the 3rd day after DSS administration (Fig. 4A,C). Moreover, hemorrhage was observed in the anus of the Con-DSS group, but not in that of the Si-DSS group. Atrophy of the large intestine was also observed in the Si-DSS group compared with that in normal mice, but the degree of atrophy was significantly suppressed compared with that in the Con-DSS group. Similarly, on the 5th day of DSS administration, atrophy of the large intestine in the Si-DSS group was suppressed compared with that in the Con-DSS group (Fig. 4B,D).

Next, structural disorders of the large intestine were examined using HE-stained samples. In the Con-DSS group, the plications of the colon were partially damaged from the 3rd day after DSS administration, and were hardly observed on the 5th day (Fig. 4E,F). In addition, the crypts of the rectum were partially vacuolated on the 3rd day, and they almost disappeared on the 5th day. Conversely, the colon and rectum in the Si-DSS group did not show any injury on the 3rd day, and only a fraction of plications and crypts were damaged on the 5th day. In addition, we conducted comparative studies based on the histological score (Table 2). As the score value of the Si-DSS group was significantly lower than that of the Con-DSS group, it was confirmed that the injury of both the colon and rectum was suppressed in the Si-DSS group (Fig. 4G,H).

**Table 2:** Histological colitis score.

Epithelial tissue disorganization		CRYPT disorganization	
**Histological colitis score**			
100%	4	100%	4
51–90%	3	51–90%	3
21–50%	2	21–50%	2
1–20%	1	1–20%	1
0%	0	0%	0

Immunocyte invasion		Erythrocyte invasion	
Large number	4	Much invasion colon tissue	4
A part	2	A few	2
A few	1	Lumen only	1
None	0	None	0

Furthermore, we performed immunostaining for F4/80 (mature macrophage marker protein) to confirm that the infiltration of immune cells into the large intestine of the Si-DSS group were less than that in the control group. In the Con-DSS group, more macrophage infiltration into the rectum was observed than in the colon (Fig. 4I,J). In the rectum, many macrophages were observed in the submucosal layer from the 3rd day after DSS treatment, and more macrophages were infiltrated in the mucosal layer in which the crypt structure was disrupted (Fig. 4I, rectum). On the 5th day, a large number of macrophage infiltrations were observed in the mucosal and submucosa layer where the epithelial cells were peeled off and the crypt structure was completely collapsed and became vacuolated (Fig. 4J, rectum). In the colon, only a few macrophages were observed in the submucosal layer on the 3rd day after DSS treatment, but on the 5th day, many macrophages were observed in the mucosal and the submucosal layer where the colon plicae had collapsed (Fig. 4I,J, colon).

On the other hand, in the Si-DSS group, only a few macrophages were observed in the submucosa of the rectum on the 3rd day after DSS treatment, and almost no macrophages were observed in the colon (Fig. 4I). On the 5th days after DSS treatment, macrophage infiltration was observed where some rectal crypt structures and colon plicae collapsed (Fig. 4J). Even on the 5th day of DSS administration, there were some areas where the rectal crypt structure and colon plicae did not collapse in the Si-DSS group, and no macrophages were observed in such areas. Therefore, the infiltration of macrophages into the large intestine of the Si-DSS group was significantly less than those of the Con-DSS group in which the colon plicae and the rectal crypt structure were almost destroyed.

From the above findings, it was clarified that administration of the Si-based agent significantly relieved damage to the large intestine by suppressing the progression of inflammation.

### Si-based agent suppressed the increase in LPO levels associated with UC

**Figure 5:**
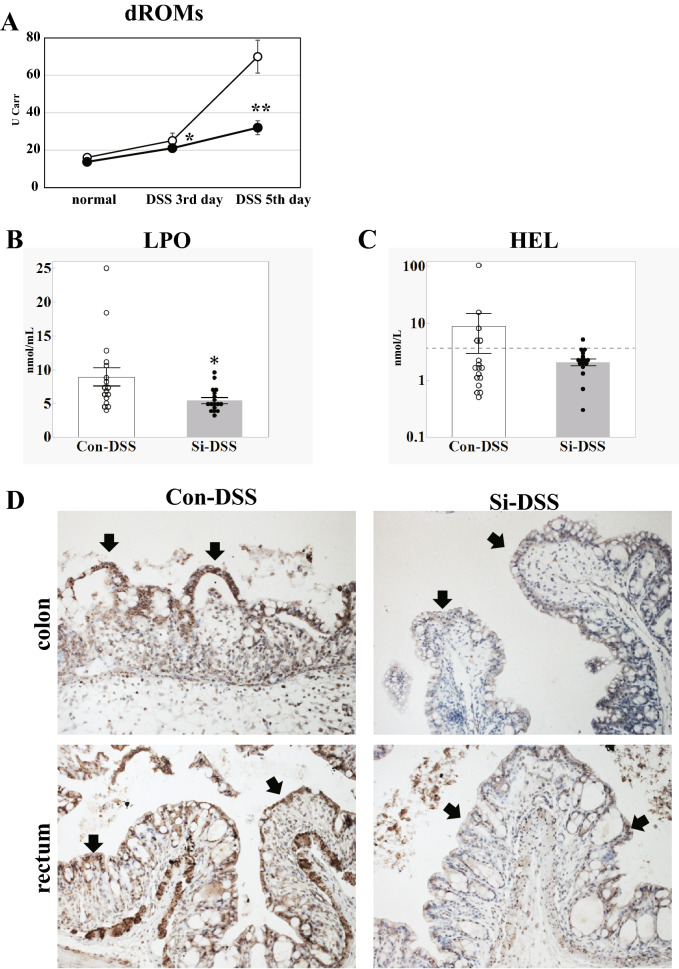
Si-based agent alleviated the oxidative stress associated with UC. Analysis of oxidative metabolite in mouse serum 5th day (**A**) and LPO in mouse serum 3rd day after 5% DSS treatment (**B**,**C**). Day-dependent changes in the mean of the dROMs value (**A**). Bar chart indicates the mean values. *LPO* lipoperoxide (**A**) and *HEL* Nε-(hexanoyl)lysine (**B**). The dot line in (**B**) indicates the detection limit for HEL (3.0). The positive rates for HEL were 29.4% (Con) and 17.6% (Si). White: Con or Con-DSS group. Black: Si or Si-DSS group. Data are expressed as mean ± SEM of 8 (**A**: normal), 9 (**A**: DSS 3rd and 5th day) and 17 mice (**B**,**C**) per group. *p < 0.05, *p < 0.05 vs. control, determined by Student’s paired *t*-test (**A**–**C**). Analysis of LPO accumulation on the 5th day after DSS administration using immunohistochemistry with anti-4-HNE antibodies (**D**). The representative photographs of the colon (upper) and the rectum (bottom) in the Con-DSS group (left) or Si-DSS group (right). Black arrows: epithelial cells. Scale bar: 50 µm.

Diacron-reactive oxygen metabolites test were performed to determine whether the Si-based agent also has an anti-oxidative effect. The dROMs value, an index of oxidative metabolites in blood, was higher after DSS administration than that in normal condition regardless of the Si-based agent administration, and it was higher on the 5th day than on the 3rd day (Fig. 5A). However, it was revealed that the increase in dROMs value of the Si-DSS group, was significantly alleviated from the 3rd day after DSS administration as compared with the Con-DSS group. No significant difference was observed between the two groups in normal dROMs value.

Next, the blood levels of LPO were analyzed with enzyme-linked immunosorbent assay (ELISA) to determine whether the Si-based agent suppresses oxidative metabolites in the blood. It was found that the blood levels of lipid hydroperoxide (LPO) in the Si-DSS group were significantly lower than those in the Con-DSS group (Fig. 5B). Moreover, the blood levels of Hexanoyl-lysine (HEL; the early LPO) of the Si-DSS group were lower than those in the Con-DSS group (Fig. 5C).

In addition, immunocytochemistry for 4-hydoxy-2-nonenal (4-HNE), an indicator of increased lipid peroxidation chain reaction, was performed to confirm that the Si-based agent alleviated the production of LPO. In the Con-DSS group, strong positive signals were observed in the cytoplasm of epithelial cells and infiltrated immune cells in the colon and rectum on the 5th day of DSS administration (Fig. 5D left panels). On the other hand, in the Si-DSS group, few positive signals were observed in the colon or rectum (Fig. 5D right panels).

These findings demonstrated that the Si-based agent suppressed LPO production through its antioxidant ability.

### Administration of the Si-based agent increased the amount of hydrogen generated in the large intestine

**Figure 6:**
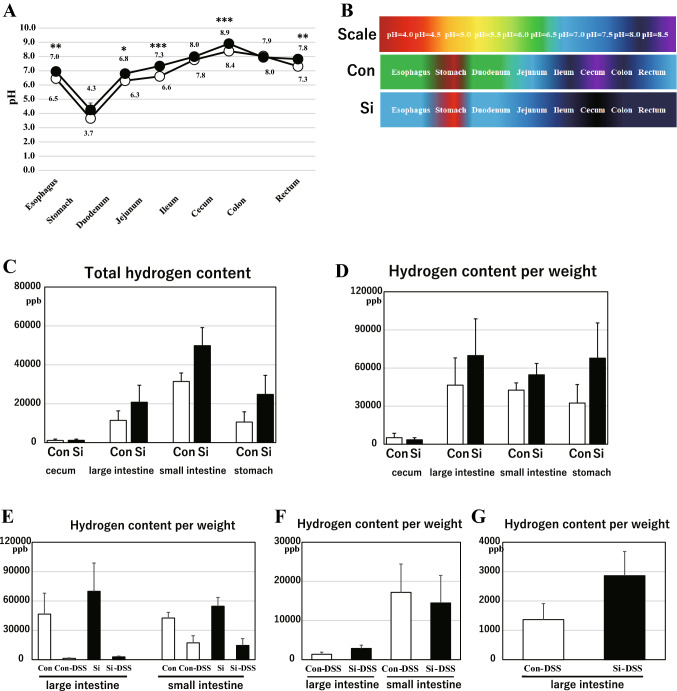
Si-based agents increased the amount of hydrogen in the large intestine. Detection of GI pH and hydrogen content. (**A**) The line graph for GI pH of the control group (white circle and dotted line) and Si group (black circle and solid line). The sections used for pH detection were the esophagus, stomach, duodenum, jejunum, ileum, cecum, colon, and rectum. Data are expressed as mean ± SEM of five mice per group. *p < 0.05, **p < 0.01, ***p < 0.001 vs. control, determined by Student’s paired *t*-test. (**B**) Each pH variation is shown as color variation based on the scale. (**C**–**G**) The average bar graphs for gastrointestinal hydrogen content of the control or control DSS group (white bar) and the Si or Si-DSS group (black bar) in the normal state (**C**,**D**) and 3 days after 5% DSS treatment (**E**–**G**). (**C**) Total hydrogen content and (**D**–**G**) hydrogen content per weight. Data are expressed as mean ± SEM of nine mice and DSS-treated mice (Con: 8, Si: 7) per group.

The results thus far revealed that the Si-based agent is effective in alleviating the symptoms of UC. Therefore, we investigated the mechanism of action of the Si-based agent in terms of antioxidant and anti-inflammatory effects. For Si-based agent to generate hydrogen, it is essential that the gastrointestinal environment is neutral or alkaline. Therefore, we measured the mouse gastrointestinal pH from the esophagus to the rectum. In the control group, the pH was neutral in the esophagus, but acidic in the stomach, weakly acidic in both the duodenum and jejunum, and finally neutral in the ileum (Fig. 6A,B). The pH of the large intestine was alkaline and that of the cecum was the most alkaline. Surprisingly, the gastrointestinal pH was generally inclined toward the alkaline side in the Si group compared with that in the control group.

Next, to examine whether hydrogen is actually generated in the intestinal tract (especially in the large intestine) of mice administered the Si-based agent, the levels of hydrogen in the stomach, small intestine, cecum, colon, and rectum were analyzed with gas chromatography. In the control group, hydrogen was detected abundantly in the intestinal tract, except in the cecum, where very slight amounts were detected (Fig. 6C). The total amount of retained hydrogen was the highest in the small intestine and similar in the large intestine and stomach. No difference was observed in the amount of retained hydrogen per body weight in the intestine other than in the cecum (Fig. 6D). In the Si-DSS group, the amount of retained hydrogen followed the same pattern as that in the Con-DSS group. Furthermore, the total amount of gastrointestinal retained hydrogen in the Si group, excluding the cecum, was higher than that in the control group. From the above-stated findings, it was clarified that the Si-based agent generated hydrogen in the intestinal tract. Interestingly, the mice affected with colitis had significantly reduced levels of hydrogen in the large intestine and small intestine, regardless of the Si-based agent treatment (Fig. 6E). Particularly, in the inflamed site, large intestine, the volume of hydrogen was significantly reduced compared with that in the small intestine (Fig. 6F,G). Surprisingly, the amount of residual hydrogen in the large intestine after the onset of colitis in the Si-DSS group was higher than that in the Con-DSS group (Fig. 6G). These results demonstrated that the decrease in the amount of hydrogen during inflammation was alleviated by the administration of Si-based agent.

### Examination of the antioxidative mechanism of the Si-based agent in relieving the symptoms of UC

**Figure 7:**
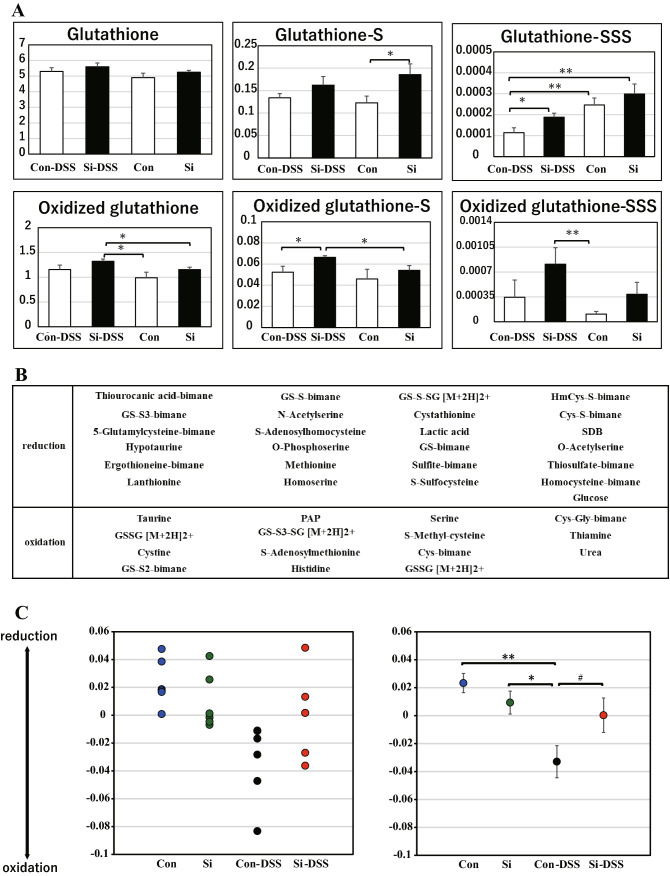
Si-based agent suppressed the intestinal oxidation associated UC via antioxidant sulfur compounds. Sulfur index analysis of the mouse large intestine. The average bar graphs for the expression of glutathione, oxidized glutathione, and each persulfide (**A**). White: control or con-DSS group; black: Si or Si-DSS group. (**B**) Contributory compounds in sulfur-index analysis. (**C**) The dot graph of individual values and the average for sulfur index analysis. Data are expressed as mean ± SEM of six mice per group. ^♯^*p* < 0.08, *p < 0.05, **p < 0.01, determined by Student’s paired *t*-test.

Si-based agent alleviate the symptoms of UC by replenishing lost hydrogen in the large intestine. It is possible that such hydrogen supplementation has anti-inflammatory and antioxidant effects. We focused on the metabolism of sulfur compounds, which are greatly involved in the redox action in the body, and conducted a comprehensive analysis of 87 types of sulfur metabolites (sulfur index analysis) in a total of four groups: the Con-DSS group, the Si-DSS group, the control group, and Si groups. As a result, 39 types of sulfur compounds were detected. The amount of glutathione-related sulfur compounds was significantly higher in the group administered the Si-based agent than in the non-administered group (Fig. 7A). Among these compounds, the expression levels of glutathione persulfide, which exhibits strong antioxidant effects, were remarkably increased. After performing multivariate analysis based on the detected sulfur-related compound data (Fig. 7B), similarity mapping analysis between groups was performed. In the control group, the normal large intestine was in the reduced state, but when colitis was induced, it shifted toward the oxidized state due to inflammation (Fig. 7C). In contrast, the large intestine of the Si group was in the reduced state similar to that of control, but the colitis-induced large intestine did not shift to the oxidized state (Fig. 7C). Taken together, we identified that the Si-based agent alleviated oxidation of the colon associated with inflammation due to colitis by inducing antioxidant sulfur compounds.

### Si-based agent alleviated the visceral pain and discomfort associated with UC

**Figure 8:**
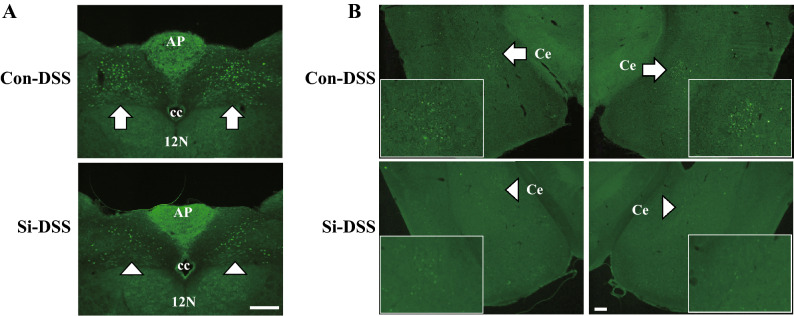
Si-based agent alleviated the visceral pain and discomfort associated with UC. Analysis of the neuronal activity influenced by colitis, using immunofluorescence staining with anti-c-Fos antibodies. (**A**,**B**) The representative photographs of the dorsal medulla (**A**) and central amygdaloid nuclei (**B**) 5 days after 5% DSS treatment. *Con* Con-DSS group, *Si* Si-DSS group. The vagal dorsal motor nuclei and solitary tract nuclei (**A**), and central amygdaloid nuclei (**B**) of a control mouse (arrow) and an Si-based agent-treated mouse (arrowhead). Square in (**B**): the central amygdaloid nuclei, 12N: hypoglossal nerve nuclei, AP: area postrema, cc: central canal, and Ce: central amygdaloid nuclei. Scale bar: 200 µm.

Psychological stress is greatly involved in remission and relapse in patients with colitis. Therefore, we also examined the effects of colitis on the brain and the therapeutic effects of the Si-based agent on the brain. To examine whether the administration of the Si-based agent alleviates the visceral pain and discomfort associated with UC, we investigated the neuronal activities in the nuclei of the dorsal medulla oblongata [the nuclei of the solitary tract (NST) and the dorsal vagal nuclei (DVN)], and the central amygdala (CeA) using immunostaining for c-Fos, a neuronal active marker. Many positive cells were observed in all analyzed nuclei of the Con-DSS group, whereas positive signals were not almost detected in the Si-DSS group (Fig. 8).

These results demonstrated that the Si-based agent alleviated the visceral pain and discomfort associated with UC, as well as the symptoms of UC.

## Discussion

In this study, the gastrointestinal symptoms, inflammation, and oxidation associated with UC were significantly alleviated by oral administration of the Si-based agent. Additionally, sulfur index analysis demonstrated that the antioxidative and anti-inflammatory effects of the Si-based agent were mediated by antioxidant sulfur compounds such as glutathione persulfide. Surprisingly, it was found that the Si-based agent not only relieved the intestinal symptoms associated with colitis but also the psychological symptoms such as visceral pain and discomfort. These findings revealed that Si-based agent could be useful therapeutic agent for UC.

Abnormal accumulation of ROS in the colon mucosa is considered to be one of UC pathogenesis^2^. It has been reported that patients with UC show increased levels of DNA oxide, enhanced ROS production in neutrophils, and decreased antioxidant mechanisms^14^. Oxidative stress is greatly involved in the onset and exacerbation of colitis, and it is likely that an appropriate antioxidant will be an effective therapeutic agent for UC. Notably, antioxidant food intake alleviated the symptoms of UC model animals^15–17^. However, superoxide and hydrogen peroxide play a physiological role in signaling molecules and regulate apoptosis, cell proliferation, and cell differentiation^18,19^. Moreover, recent studies have suggested that an excess of antioxidants inhibits essential intravital defense mechanisms, thus increasing mortality and cancer incidence^20,21^. We believe that hydrogen can overcome such shortcomings of antioxidant treatment. Hydrogen specifically neutralizes only harmful hydroxyl radicals, and does not affect superoxide or hydrogen peroxide^8^. To the best of our knowledge, no side effects have been reported to date^22^. Moreover, hydrogen has been shown to be effective in alleviating the symptoms of various oxidative stress-related diseases model animals containing UC^9,23,24^. Although the hydrogen molecule is an excellent antioxidant, there is still some scope for improvement in its administration method. The main methods of administration are inhalation of hydrogen gas and oral administration of hydrogen-rich water. As hydrogen gas is an explosive gas and dangerous to use, it can be used only in limited places such as hospitals. Furthermore, hydrogen is only slightly soluble in water, and the dissolved amount cannot be maintained owing to its permeability. Thus, an administration method that can sustain a large amount of hydrogen continuously in vivo is essential.

We succeeded in developing an Si-based agent that could continuously generate a large amount of hydrogen for a long time when suspended in water^11^. In addition, Si-based agent reportedly generate more hydrogen in weakly alkaline solution than in neutral water. It was expected that oral administration of the Si-based agent would produce a large amount of hydrogen in the small intestine and large intestine. Our findings demonstrated that the mouse intestinal pH was neutral to weakly alkaline from the duodenum to the rectum, and the amount of hydrogen contained in the large intestine was increased by administration of the Si-based agent (Fig. 6). As the Si-based agent filled the intestinal tract with a large amount of hydrogen, it was considered that it could solve the problem of in vivo administration of hydrogen. Because LPO production, which is the primary cause of worsening of UC symptoms, was significantly suppressed by the Si-based agent (Fig. 5), constant filling with hydrogen in the large intestine is highly effective in alleviating the symptoms of colitis. Surprisingly, the onset of colitis significantly reduced the gastrointestinal hydrogen content. Because it has been reported that the levels of ascorbic acid, vitamin E, and β-carotene decrease in patients with IBD^25,26^, the hydrogen were thought to be excessively consumed in response to oxidative stress induced by UC. As described above, the amount of hydrogen in colitis is extremely low, regardless of administration of the Si-based agent, compared with that in the normal state. However, the reduction in hydrogen content in the large intestine was smaller in the Si-DSS group than in the Con-DSS group (Fig. 6E–G). Interestingly, the reduction in hydrogen in the small intestine was similar in both groups. That is, the residual amount was different only at the inflamed site. This difference in the residual amount of hydrogen at the site of inflammation might lead to the alleviation of the symptoms of colitis by the Si-based agent. These results might have contributed to symptom relief and an increase in the number of survival days (Fig. 1). It has been shown that Si-based agent could be very effective therapeutic agent for the antioxidative treatment of UC. In the UC model, hydrogen sulfide, which shows increased levels in the intestinal tract with inflammation, stimulates cystine production and increases glutathione levels^27^. Glutathione, an antioxidant, reduces oxidative stress by neutralizing ROS in the large intestine^28^. Administration of the glutathione precursor acetylcysteine relieved the symptoms of UC model mice^29^. Thus, sulfur metabolism is closely related to the degree of symptoms of UC. In fact, it was revealed that the normal large intestine was in the reduced state on the basis of sulfur metabolism, but the large intestine with UC model mice was in the oxidized state (Fig. 7C). That is, the large intestine with colitis was in a state of intense oxidative stress. However, the oxidation associated with colorectal inflammation was suppressed by administration of the Si-based agent. This result was considered to be partly due to the increase in the levels of the reactive sulfur species (RSS) (Fig. 7A). The RSS are compounds with a high nucleophilic reactivity due to the addition of an excess of sulfur atoms to a thiol group^30–34^. In our study, there were no significant differences in the expression levels of reduced or oxidized glutathione, regardless of administration of the Si-based agent; however, the expression of RSS, such as glutathione trisulfide, oxidized glutathione trisulfide, glutathione sulfide, and oxidized glutathione sulfide, was increased in the Si-DSS group. It has been reported that RSS exhibit not only high nucleophilicity but also strong antioxidant activity and oxidative stress resistance compared with ordinary thiol compounds such as glutathione and cysteine. In particular, the antioxidant effect of this sulfur persulfide became stronger in proportion to the number of sulfur atoms added. Therefore, the increase in the trisulfide amount seemed to contribute to the strong antioxidant effect of the Si-based agent. In addition, glutathione and GPx decompose LPO into lipid alcohols^35,36^. LPO is a major factor that exacerbates the symptoms of UC, and detoxification of LPO is important for symptom relief. Since the expression levels of oxidized glutathione trisulfide in the Si-DSS group were higher than those in the Con-DSS group, the GPx system was considered to be enhanced (Fig. 7A). In fact, the amount of the serum oxidative metabolite such as LPO in the serum and the LPO accumulation in the colonic epithelial cells was significantly reduced in the Si-DSS group (Fig. 5). In summary, in the Si-DSS group, the colon was thought to be protected from oxidative stress by detoxification of LPO via enhancement of the glutathione/GPx system.

DSS is a mucopolysaccharide that causes intestinal mucosal epithelial damage. After mucosal barrier disruption, DSS is phagocytosed by antigen-presenting cells (APCs) in the submucosal layer. T cell activation by APCs induces symptoms similar to those in patients with IBD^37^. This shows that the DSS-induced UC model was generated in response to inflammation; thus, it is possible that the Si-based agent might also have anti-inflammatory effects^38^. In fact, the Si-based agent suppressed the symptom associated with inflammation (Figs. 2, 3, 4, 5). Anti-inflammatory effect of the Si-based agent might be related to the hydrogen produced from the Si-based agent. Hydrogen itself has been reported to have anti-inflammatory effects in inflammatory diseases caused by various systemic inflammatory inducers^25^—concanavalin A-induced hepatitis^10^, DSS-induced UC^9^, and LPS-induced sepsis^39^. Hydrogen exerts anti-inflammatory effect by suppressing the expression of factors involved in promoting inflammation, such as NF-κB, TNF-α, IL-6, CCL2, and INF-γ^40,41^. Moreover, we also observed an increase of the RSS expression in the colonic epithelium of the Si-DSS group. RSS with polysulfides reportedly protect cells from inflammation by negatively regulating the TLR4-mediated pro-inflammatory signaling pathway^42^. From the above findings, it was presumed that the anti-inflammatory action of the Si-based agent was mediated by two mechanisms: a direct anti-inflammatory action via the generated hydrogen and an indirect anti-inflammatory action through the induced RSS.

UC is an intractable disease with repeated remission and relapse. Since inflammation of the large intestine affects brain function through brain–gut interaction, patients with colitis are at a high risk of developing psychiatric disorders, such as depression and anxiety, during the exacerbation period^43,44^. Psychiatric symptoms are mainly associated with relapse^45,46^. In fact, psychologically or physically stressed rodents exhibit worse symptoms of colitis^47–50^. A vicious cycle of colorectal inflammation and psychiatric symptoms makes it difficult to completely cure colitis. For the complete cure of UC, it is important to treat not only the large intestine, which is the affected area of UC, but also the brain, as it is involved in relapse. Our Si-based agent also succeeded in alleviating the effect on the brain at the onset of UC. Visceral discomfort and pain induced by UC is transmitted to DVN and NST by vagus nerve^51^. Moreover, Unpleasant visceral stimulation has been reported to increase the expression of c-Fos in the CeA, the emotional center^52,53^. Five days after DSS treatment, the number of active neuronal cells in the DVN, NST, and CeA increased in the brain of the Con-DSS group, revealing that the control mice felt extensive visceral discomfort (Fig. 8). In contrast, in the Si-DSS group, the number of activated neurons was significantly reduced. This meant that the visceral discomfort due to UC was significantly reduced in the Si-DSS group compared with that in the Con-DSS group. If the inflammation of the large intestine subsides, but the visceral discomfort persists, the inflammation is likely to recur. In fact, patients with irritable bowel syndrome (IBS), a typical bowel disease in which stress is associated with gastrointestinal symptoms, reportedly had more than 16-fold increase in the incidence of IBD^54^. Therefore, relieving mental stress is particularly important in UC. Si-based agent can alleviate inflammation and oxidative stress in organs other than the intestinal tract at the hydrogen generation site, such as that in Parkinson's disease and renal failure^12^. Therefore, Si-based agent are considered excellent therapeutic agent for symptom relief and sustained remission of UC.

Here, we showed that Si-based agent alleviate the symptoms of UC model mice through antioxidant and anti-inflammatory effects. Finally, we examined the shortening of the large intestine of UC model mice treated with various concentrations (from 0.025 to 2.5%) of Si-based agent to evaluate its efficacy (Supplementary Fig. S1). It was revealed that administration of a Si-based agent at a concentration of 1% or more stably alleviated the shortening of the large intestine. Surprisingly, some mice were also observed to alleviate the shortening with low concentrations such as 0.025%. These findings suggested that once approved as a pharmaceutical agent, the Si-based agent could be highly expected to help alleviate the symptoms of UC because the range of efficacy of the Si-based agent was very wide. However, in clinical practice, subsidence of inflammation is not a complete cure. As long as the patient experiences pain and discomfort, the illness continues. Since it was difficult to measure pain or unpleasant sensation in mice, we assessed the recovery status by examining the decrease in nerve activity related to visceral pain and discomfort. In addition, the degree of inflammation of the mouse intestinal tract was determined by MRI analysis, a technique often used in clinical practice. With these two approaches, we were convinced that the Si-based agent could be a highly effective therapeutic agent for symptom relief and sustained remission of UC in clinical practice in the future.

## Methods

### Animal models

7-week-old male C57BL/6J mice were purchased from Japan SLC (Shizuoka, Japan). UC models were produced by administering drinking water containing 5% DSS to mice. The mice were kept at a constant temperature (23–25 °C) and fed with a custom diet and water ad libitum. The intake of diet and drinking consumed per day was measured for each cage (3 mice per cage) during a following period: each diet treatment for a week, DSS treatment for 3 days or 5 days after each diet treatment for a week. All experiments with mice were performed according to the protocols approved by the Committee of Animal Experiments of Osaka University (approval number 02-001-003), in accordance with the National Institutes of Health Guide for Care and Use of Laboratory Animals. All efforts were made to minimize the number of mice used and decrease animal anguish. This research was conducted in compliance with the ARRIVE guidelines.

### Si-based agent-containing diet

The Si-based agent was produced from Si powder (Osaka Titanium technologies Co., Ltd. Si 4N Powder, < 300 µm). The powder was sifted and Si powder of sizes less than 45 µm was obtained. Then, Si nanopowder was produced by use of the beads milling method, as previously described^12^. We prepared custom diets: 2.5% Si-based agent containing AIN93M, and only AIN93M as the control (Oriental Yeast Co., Ltd., Tokyo, Japan). Each diet was administered one week before the start of DSS administration.

### Colitis score and histological score

The revised versions of both scores of Kajiya et al*.* were used^9^. We blindly evaluated for both. For the colitis score, the weight loss rate, stool hardness, and anal bleeding were quantified as shown in  Table 1. Weight loss was divided into five categories based on the degree of reduction rate. Stool hardness and anal bleeding were classified into three categories according to the following criteria: Stool hardness was classified according to the shape of the stool (shaped stool, unformed stool, and liquid stool) and the presence or absence of stool adhesion to the anus, and anal bleeding was classified according to the degree of bleeding (presence, absence, and slight adhesion). The average value of the numerical values of the above three items was used as the colitis score. The colitis score was 0 when healthy and 4 when colitis was maximally active.

For the histological score, the epithelial tissue disorganization, crypt disorganization, immunocyte invasion, and erythrocyte invasion were quantified as show in Table 2. The epithelial tissue disorganization and crypt disorganization were divided into five categories according to the degree of disorganization of each tissue. Immunocyte invasion and erythrocyte invasion were divided into five categories according to the degree of invasion in the large intestine. Each item in the histological colitis score was 0 when healthy and 4 when colitis was maximally active.

### Magnetic resonance imaging (MRI) and magnetic resonance angiography (MRA)

In vivo MRI of mice was conducted using an 11.7 T vertical bore scanner (AVANCE II 500WB; Bruker BioSpin, Ettlingen, Germany). Anesthesia was initially induced with 2.0% isoflurane and maintained with 1.6% isoflurane during MRI. The body temperature of the mice was maintained at 37 °C with circulating warm water. T_2_ weighted axial and sagittal images with fat suppression were obtained using the rapid acquisition with relaxation enhancement (RARE) technique. The acquisition parameters were as follows: field of view (FOV) = 25 mm × 25 mm, matrix size = 256 × 256, in-plane resolution = 98 μm, slice thickness = 500 μm for axial images and 300 μm for sagittal images, repetition time (TR) = 6000 ms, echo time (TE) = 36.7 ms, number of averages (NA) = 8, and acquisition time (TA) = 13 min. MRA was performed using the 2D time-of-flight (TOF) technique^55^. The acquisition parameters were as follows: FOV = 25 mm × 25 mm, matrix size = 256 × 256, in-plane resolution = 98 μm, slice thickness = 400 μm, slice pitch = 250 μm, TR = 20 ms, TE = 2.7 ms, NA = 4, and TA = 16 min. The volume rendering method was performed with the acquired images to visualize blood vessels three dimensionally. High signal intensity regions in the obtained images indicated the inflammation of colon. The pixels of inflamed edematous regions with high signal intensity in the axial image were measured using the Image J 1.52a software (National Institutes of Health, USA), and a comparative analysis between the control group and Si-based agent-treated group was conducted.

### Measurement of the large intestine length, and hematoxylin and eosin (HE) staining, and immunohistochemistry

Under deep anesthesia using a combination anesthetic (0.3 mg/kg medetomidine, 4.0 mg/kg midazolam, and 5.0 mg/kg butorphanol)^56^, DSS-induced colitis mice were perfused with 4% paraformaldehyde (PFA) in 0.01 M phosphate buffer (PB, pH 7.4). The large intestine (from the cecum to anus) was removed and post-fixed with the same fixative. After the length from the colon to the rectum was measured using a ruler, the colon samples were washed in 0.1 M phosphate-buffered saline (PBS). After dehydration in a series of ascending ethanol concentrations (70%, 80%, 90%, and 100% ethanol), the samples were embedded in paraffin (tissue preparation, T580, FALMA, Tokyo, Japan) using Clear Plus (FALMA). The paraffin-embedded colon samples were sliced into 7 µm sections using a microtome (RM2145, Leica Microsystems K.K, Tokyo, Japan) and then mounted on glass slides (Matsunami-glass, Osaka, Japan). After deparaffinization, samples were stained with the HE solution (FUJIFILM Wako Chemicals Corporation, Osaka, Japan) or immunostained for 4-HNE (antigen retrieval treatment), F4/80 (no antigen retrieval treatment) as follows. Before blocking with 3% bovine serum albumin (BSA)-containing 0.1MPBS, antigen retrieval treatment with 0.01 M citrate buffer (pH6.0) at 95 °C for 40 min was performed. The colon samples were treated with anti-4-HNE mouse monoclonal antibody (Japan Institute for the Control of Aging, Nikken SEIL Co. Ltd., Shizuoka, Japan) or anti- F4/80 rat monoclonal antibody (abcam, Cambridge, UK) in the blocking solution at 4 °C overnight. After washing in 0.1 M PBS thoroughly, the samples were incubated with biotin-conjugated anti-mouse immunoglobulin G (IgG) antibody (4-HNE: Vector Laboratories, Inc. Burlingame, CA) or biotin-conjugated anti-rat IgG antibody (F4/80: Vector Laboratories) in 0.1 M PBS at 22 ± 2 °C for 30 min. The signal amplification with avidin–biotin complex was followed by the visualization with 50 mM Tris-buffered saline (TBS; pH 7.4) containing 1.25% 3,3′-diaminobenzidine (DAB: Merck KGaA, Darmstadt, Germany) and 0.75% hydrogen peroxide. After stop of reaction using 50 mM TBS, the samples were counter-stained with hematoxylin solution. Finally, all stained slides were dehydrated, coverslipped and then mounted with Entellan (Merck KGaA). All samples were analyzed using a light microscope (BX53; Olympus Corporation, Tokyo, Japan).

### Immunofluorescence staining

Immunofluorescence staining was performed as previously described^57^. After perfusion with 4% PFA in 0.1 M PB, the brains of DSS-induced colitis mice were removed and then treated with the same fixative. The fixed brains were cryoprotected in 0.1 M PB containing 30% sucrose, frozen in dry ice, and then cut into slices. The brain sections were floated in 0.01 M PBS and stored at 4 °C until use. After treatment with the blocking solution (0.3% Triton-X and 3% BSA in 0.01 M PBS) to block non-specific staining and increase permeability to antibodies, the brain sections were incubated with anti-c-Fos rabbit polyclonal antibodies (abcam) in the blocking solution at 4 °C overnight. After washing several times in 0.01 M PBS, the samples were treated with anti-rabbit IgG antibodies (Thermo Fisher Scientific) in 0.01 M PBS at 22 ± 2 °C for 1 h. The samples were washed thoroughly in 0.01 M PBS, followed by mounting on slides using PermaFluor (Thermo Fisher Scientific). The stained samples were analyzed using a BX53 microscope and a confocal microscope (BX61 type FV1000D, Olympus Corporation).

### Quantitative reverse transcription-polymerase chain reaction (qRT-PCR)

According to the protocol, total RNA was extracted from the large intestine with TRIzol (Life Technologies Japan Ltd., Tokyo, Japan) using a Dounce Tissue Grinder (DWK Life Sciences, LLC, NJ, USA), and 500 ng of RNA was reverse transcribed to complementary DNA using the Super Script III First-Strand Synthesis Super MIX kit (Life Technologies Japan Ltd.). qRT-PCR analysis was conducted using ABI Power Up SYBR Green Master Mix on ABI Quant Studio7 (T Life Technologies Japan Ltd.). The qRT-PCR primer sets for cDNA amplification were as follows: TNF-α (forward: 5′-TCCAGGCGGTGCCTATGT-3/reverse: 5′-CACCCCGAAGTTCAGTAGACAGA-3), IL-6 (forward: 5′-CTGCAAGAGACTTCCATCCAGTT-3′/reverse: 5′-AAGTAGGG AAGGCCGTGGTT-3′), IFN-γ (forward: 5′-TCAAGTGGCATAGATGTGGAAGAA-3′/reverse: 5′-TGGCTCTGCAGGATTTTCATG-3′), CXCL2 (forward: 5′-CTCAGTGC TGCACTGGTCCTG-3′/reverse: 5′-CTGGGGGCGTCACACTCAAG C-3′), and GAPDH (forward: 5′-CCTCGTCCCGTAGACAAAATG-3′/reverse: 5′-TCTCCACTTT GCCACTGCAA-3′). Data are presented as the mean of three comparative cycle thresholds. The cycle threshold value of each RNA was normalized by subtracting the cycle threshold value of GAPDH RNA.

### Oxidative stress measurement

Under deep anesthesia, whole bloods were collected from the right atrium of the following group: each diet treatment for a week, DSS treatment for 3 days or 5 days after each diet treatment for a week. The samples were centrifuged (3000 rpm, 10 min, 4 °C) and serum were collected. The serum samples were stored at − 80 °C until use. To examine the serum levels of ROS metabolites, the levels of dROMs were measured by REDOXLIBLA (Wismerll Co. Ltd., Tokyo, Japan). The results of dROM test were shown as arbitrary units (U. Carr). 1 U Carr corresponds to 0.8 mg/L of hydrogen peroxide^58^.

### LPO detection

Serum samples were obtained from supernatants by the centrifugation of blood collected from the heart. HEL and LPO levels were measured using an HEL enzyme-linked immunosorbent assay (ELISA) kit and LPO assay kit (Japan Institute for the Control of Aging, Nikken SEIL Co. Ltd.), respectively.

### pH detection

After deep anesthesia, the GI was removed rapidly and broken into eight sections: the esophagus, stomach, duodenum, jejunum, ileum, caecum, colon, and rectum. After removing each gastrointestinal section, the GI pH was measured directly using pH test strips (Advantec Toyo Co. Ltd., Tokyo, Japan).

### Gas chromatography

After deep anesthesia, various tissues (cecum, colon including rectum, small intestine, and stomach) were removed, quickly inserted into a glass tube for gas chromatography, and allowed to stand for 20 min. One milliliter of air in the tube was collected and injected into a TOA DKK-TOA DH-35A portable dissolved hydrogen meter. In addition, the air samples collected in the laboratory and the analysis room were analyzed at the start and end of the experiment, and the average of all the measured values was used as the cutoff value. For the analytical value (parts per billion [ppb]) of each organ, the value corrected with the cutoff value was adopted as the hydrogen volume contained in each tissue.

### Sulfur index analysis (sulfur metabolomics)

Sulfur index analysis was carried out using *S*-bimanyl derivatives through LC MS/MS (Shimadzu Nexera UHPLC system with on-line LCMS 8040, Kyoto, Japan), as previously described^59–62^. In brief, the sulfur compounds were extracted from colon samples by the addition of methanol and then fluorescently derivatized using a thiol-specific alkylating reagent (mono-bromobimane). Levels of the target metabolic substances were calculated from the peak area, by using mass chromatography and indicated as relative amounts following normalization using the internal standard (d-camphor-10-sulfonic acid) peak area. In addition, mapping analysis of similarity between samples based on multivariate analysis using the analytical values of the detected sulfur-related compounds was performed using the R software package.

### Statistical analysis

For all studies, Student’s *t*-test or Wilcoxon rank sum test was performed to compare the differences between two groups. The results of statistical analysis were considered significant at ^†^*p* < 0.09, ^††^*p* < 0.08, *p < 0.05, **p < 0.01, and ***p < 0.001. The results are expressed as mean ± SEM.

## Supplementary Information

Supplementary Information.

## Data Availability

All relevant data are within the paper and its Supplementary Information files.
