# Author Correction: A new therapy against ulcerative colitis via the intestine and brain using the Si-based agent

**DOI:** 10.1038/s41598-022-19609-3

**Published:** 2022-09-07

**Authors:** Yoshihisa Koyama, Yuki Kobayashi, Ikuei Hirota, Yuanjie Sun, Iwao Ohtsu, Hiroe Imai, Yoshichika Yoshioka, Hiroto Yanagawa, Takuya Sumi, Hikaru Kobayashi, Shoichi Shimada

**Affiliations:** 1 Department of Neuroscience and Cell Biology, Osaka University Graduate School of Medicine, 2-2 Yamadaoka, Suita, Osaka 565-0871 Japan; 2 Addiction Research Unit, Osaka Psychiatric Research Center, Osaka Psychiatric Medical Center, Osaka, 541-8567 Japan; 3 SANKEN, Osaka University, Osaka, 567-0047 Japan; 4 University of Tsukuba, Faculty of Life and Environmental Sciences, 108-2, Cooperative Research Building A, Ibaraki, 305-8577 Japan; 5 Euglena Co., Ltd., Tokyo, 408-0014 Japan; 6 University of Tsukuba, R&D Center for Tailor-Made-QOL, 108-2, Cooperative Research Building A, 1-1-1 Tennodai, Tsukuba, Ibaraki 305-8577 Japan; 7 Graduate School of Frontier Biosciences, Osaka University, Osaka, 565-0871 Japan; 8 Center for Information and Neural Networks, National Institute of Information and Communications Technology (NICT) and Osaka University, Osaka, 565-0871 Japan; 9 Institute for Open and Transdisciplinary Research Initiatives, Osaka University, Osaka, 565-0871 Japan; 10 Department of Cell Biology, Graduate School of Medicine, Osaka University, Osaka, 565-0871 Japan

Correction to: *Scientific Reports* 10.1038/s41598-022-13655-7, published online 10 June 2022

The original version of this Article contained errors in Figure 7A, where the X-axis labels of the bar graphs were incorrectly labelled as ‘Con’, ‘Si’, ‘Con-DSS’ and ‘Si-DSS’ (from left to right). The labels have now been corrected to ‘Con-DSS’, ‘Si-DSS’, ‘Con’ and ‘Si’ (from left to right).

The original Figure 7 and accompanying legend appear below.

**Figure 7:**
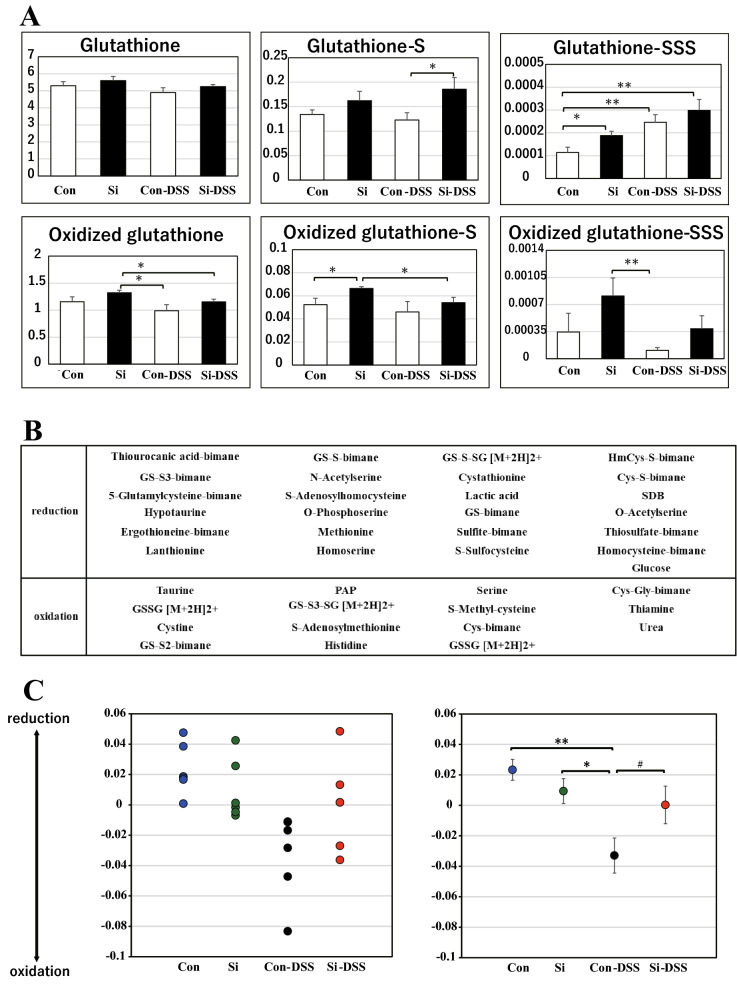
The caption to be typeset alongside it: Si-based agent suppressed the intestinal oxidation associated UC via antioxidant sulfur compounds. Sulfur index analysis of the mouse large intestine. The average bar graphs for the expression of glutathione, oxidized glutathione, and each persulfide (**A**). White: control or con-DSS group; black: Si or Si-DSS group. (**B**) Contributory compounds in sulfur-index analysis. (**C**) The dot graph of individual values and the average for sulfur index analysis. Data are expressed as mean ± SEM of six mice per group. ^♯^*p* < 0.08, *p < 0.05, **p < 0.01, determined by Student’s paired *t*-test.

The original Article has been corrected.

