# Supplementary material for: A new therapy against ulcerative colitis via the intestine and brain using the Si-based agent
Source: Sci Rep. 2022 Jun 10;12:9634. doi: 10.1038/s41598-022-13655-7 (PMC9187638; doi:10.1038/s41598-022-13655-7)
Supplement: Supplementary file 1 — Supplementary Information. [file 41598_2022_13655_MOESM1_ESM.pdf]

Fig. S1

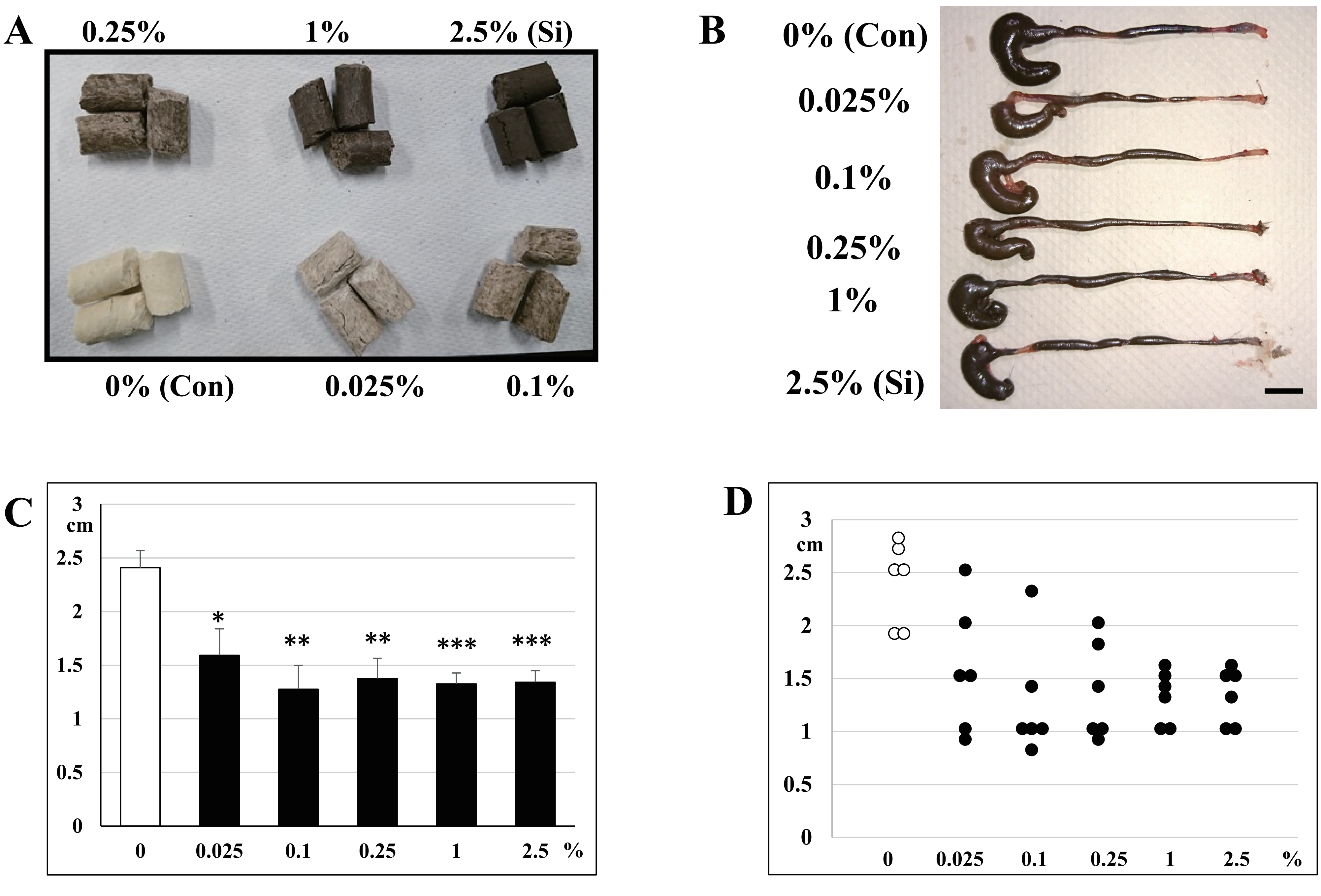
A
0.25%
1%
2.5% (Si)
0% (Con)
0.025%
0.1%
B
0% (Con)
0.025%
0.1%
0.25%
1%
2.5% (Si)
C
3
cm
2.5
2
1.5
1
0.5
0
*
**
**
***
***
0
0.025
0.1
0.25
1
2.5
%
D
3
cm
2.5
2
1.5
1
0.5
0
0
0.025
0.1
0.25
1
2.5
%

Fig. S1 The Si-based agent at a concentration of 1% or more stably alleviated the s hortening of the large intestine. Measurement and pathological analysis of the large int estine of 5% DSS-treated mice. The degree of shortening was defined as the value ob tained by subtracting the measured value of the length of each colitis mouse from the av erage measured value of the length of the large intestine of normal mice. (A, B) The repr esentative photograph of the various concentration of Si-based agent-containing diet (A) and the large intestine of mice treated with each diet 3days after DSS treatment (B). (C, D) The average bar graph shows the shortening values 3days after DSS treatment (C) an d the dot graph indicates the individual values (D). white: 0% (Con)-DSS group, and bla ck: various concentrated Si-DSS groups (0.025%, 0.1%, 0.25%, 1% and 2.5%). Scale ba r: 1cm (B). Data are expressed as mean ± SEM. *$p < 0.05$, **$p < 0.01$ and ***$p < 0.001$ vs. 0% (Con)-DSS, determined by Student's paired *t*-test.
